# Supplementary material for: The interactome of intact mitochondria by cross-linking mass spectrometry provides evidence for coexisting respiratory supercomplexes
Source: Mol Cell Proteomics. 2017 Dec 8;17(2):216–32. doi: 10.1074/mcp.RA117.000470 (PMC5795388; doi:10.1074/mcp.RA117.000470)
Supplement: Supplemental Data [file supp_RA117.000470_133922_0_supp_23978_fzffwf.zip › spectra_annotation/mito_spectra_annotation/253-1-1-1-19-1.pdf]

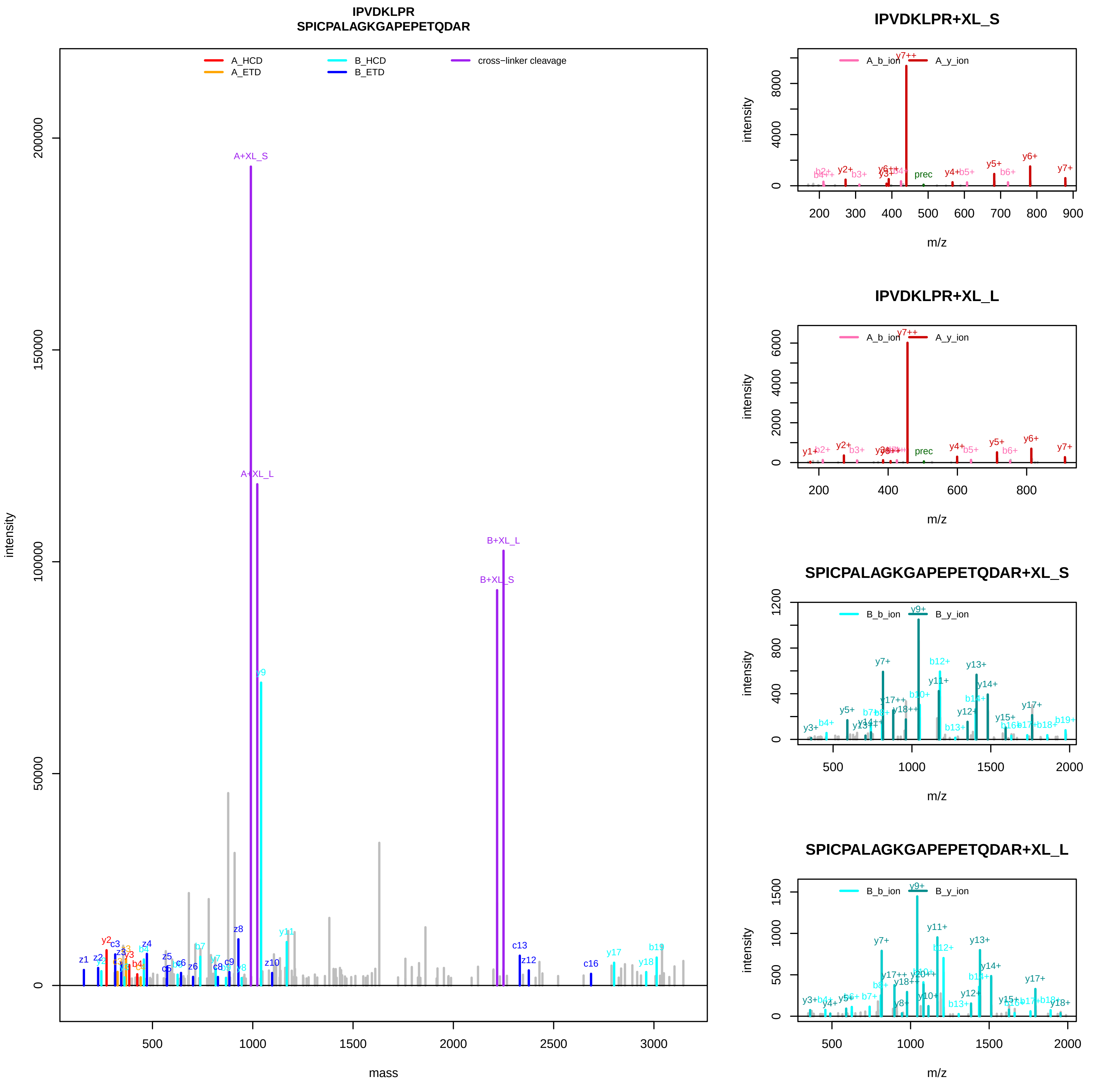
IPVDKLPR
SPICPALAGKGAPEPETQDAR
A_HCD
A_ETD
B_HCD
B_ETD
cross−linker cleavage
A+XL_S
A+XL_L
B+XL_L
B+XL_S
intensity
mass
0
50000
100000
150000
200000
500
1000
1500
2000
2500
3000
z1
z2
y2
c3
z3
y3
b4
z4
b4
z5
c5
c6
z6
b7
y7
c8
c9
z8
y8
z10
y9
y11
c13
z12
c16
y17
y18
b19
IPVDKLPR+XL_S
A_b_ion
A_y_ion
y7++
y2+
y6+
y5+
y7+
y4+
b5+
b6+
b3+
prec
m/z
IPVDKLPR+XL_L
y1+
b2+
y2+
b3+
prec
y4+
b5+
y5+
b6+
y6+
y7+
SPICPALAGKGAPEPETQDAR+XL_S
B_b_ion
B_y_ion
y9+
y7+
b12+
y13+
y11+
y14+
b10+
y17++
y18++
y5+
b4+
y3+
y12+
b13+
y15+
y17+
b19+
SPICPALAGKGAPEPETQDAR+XL_L
y9+
y11+
y7+
b12+
y13+
y14+
y17+
y12+
b13+
y15+
y18+
y10+
y8+
y3+
y4+
b7+
